# The Wear and Tear of Medical Life

**Published:** 1861-01

**Authors:** 


### Art. VII.—THE WEAR AND TEAR OF MEDICAL LIFE.

An Indian officer who has travelled over a large portion of the habitable globe, remarked the other day, that in whatever quarter of the world you may happen to alight, there you are certain to meet with a British medical man. In the remote islands of the Indian Archipelago, as well as in the backwoods of the Far West, an English doctor never fails to present himself! They are, happily for the human race, spread like a fan all over the earth. The smallest colony has often more than one. In the most unfrequented localities you find an English physician or surgeon settled as a permanent resident. Ask in the hour of difficulty and danger for a doctor, and, sesame! a son of Great Britain stands by your side! How is this? What inducement can have led the adventurous student, or the experienced practitioner, to migrate so far from his native land? What a history of individual chances, hopes, fears, shame, or necessity, must it not be that can have driven or seduced the well-educated, if not the well-connected and ambitious aspirant, from the grand arena of the social world, where merit is rightly rewarded, wealth procured, and a name achieved! What destiny has provided for the numberless outcasts of mankind, "remote from consequence, and unknown to fame," to be thus skilfully cared for in the season of sickness, the day of calamity, and the hour of death? What a boon, and how little dreamed of in this huge metropolis, overwrought with the business of life, and merged in the vortex of headlong egotism or inexorable toil!

But it is part of the nature of the Saxon race to migrate from their home. It belongs to that peculiar spirit which rules the progress of our "Ocean Isle," to look beyond its shores, and to wend its way to the most distant climes, never more to return, perchance, to the spot from which it sprang. Of the numbers who pass their examinations weekly at the several medical boards, how few, comparatively speaking, ever settle down to practise in their native land! The greater number are never heard of again from the hour in which they obtain their diplomas to that in which their names are inserted in the obituary of some local or more widely circulated newspaper. Perhaps even this last mark of respect is denied them; and they finish their days in some distant clime alone, unknown, and unregretted even by those with whom they started in the morning of their prime. Premature disease, contracted, perhaps, during the period of their studies, cuts off not a few; the ocean or the desert swallows up others; disappointment and the icy hand of penury and want stifle many a fine genius and worthy soul; while the army, navy, and the mercantile marine might tell the twice-told tale of all the rest. A remnant remains at home. Of this remnant, alas! but a small section surmounts the stern obstacles raised up against their strenuous efforts at advancement; and, out of the whole number, a few—a very few—survive to gain a satisfactory competence, or to rise to distinction in their profession.

Do you see yonder lean man, grey-headed and intelligent, walking down St. James's-street, clad in rusty black, and gazing at you with his cold, grey eye, as you quickly pass him by? Ambition, fatal ambition, marked him for her own. He came from a provincial town where he was well known, and, with every legitimate diploma in his pocket, duly signed, sealed, and delivered by the examining authorities, started in the great race to compete the prize among the millions of the modern Babylon. Ah, foolish effort! "Dark was his morn of life, and bleak the spring." The great world passed him by, and heeded not the talents buttoned up within his vest, nor heard the cry of his young ones pining away in a large house in a fashionable quarter of the town. He has grown grey in quest of a reputation perpetually fleeting from his sight, and eluding his tremulous grasp.

In the back room of a paltry lodging, in the deserted street of a fashionable watering-place, stretched on a water-bed, palsied and dying, lies one who not long ago resided in London, sat in professional state to receive the numerous patients that beset him daily, or rolled along the streets in his chariot to keep his appointments with due propriety and punctuality. How jaunty was he then! How little did he foresee the inroads that would be made upon the slender texture of his nerves by the constant excitement of the mind, the broken nights, the anxious days, and the precarious tenure of maintaining professional infallibility at the top of its bent. And then he wrote a book. The book sold, and extended his reputation, and added to his toil. He wrote a second, and the second sold as well as the first. He was at the acme of his hopes. A London celebrity was before him, when, lo! his eyesight failed in the zenith of his years, and he withdrew to linger out his term on a small pittance in blind obscurity. Adversity has no friends. The crowd that had hung attentive on his lips, forgot that he had ever existed, and passed on to the adulation of their next new idol.

And this aged gentleman, grey headed and shabby genteel, with wisdom on his brow and discretion in his gait—how is it that this venerable epitome of medicine has never plucked a leaf from the golden bough of fame, nor taught the public to credit what he really is—an able, a learned, and a useful man? But such are the chances of the medical life. The pretender too often carries the day; and loud assertion and bombast prevail over solid merit, modesty, and truth.

The pursuit of medicine, however, is not to be blamed for casualties such as these. Most likely they are the lot of humanity, and belong to that class of severe teaching to which we, as moral beings, are subjected in the pilgrimage of life. Success is a rare result. The most gifted are not always the most successful. It belongs to a happy combination of things, over which we have no control, to be able to bring forward and produce before the world, in their proper light, those high qualities with which we have been endowed, and to offer them for general service in the most agreeable and acceptable manner. The kindest of patrons can do little or nothing for us, unless we can do much for ourselves; and the most fortunate concurrence of events is fruitless without moderation, sagacity, and the consummate perception of time, circumstance, and place.*

In the routine of life, the practice of medicine affords a subsistence to about thirty thousand medical men in this country. Perhaps the average incomes of the whole taken together range from 500*l.* to 700*l.* a year. The majority are general practitioners; a class of men endued with a comprehensive and strictly practical education. The health and safety of the million are in their keeping. The sanitary management of private families is regulated by them. The unions, the workhouses, the provincial dispensaries, and hospitals, are officered by them. By them, the poor are waited on in their own dwellings; and a vast amount of disease is attended, alleviated, or cured, by their unpaid, if not unrewarded ministrations. The public are scarcely alive to the fact of how much the health of the community at large is in their hands, and how conscientiously and ably they discharge their duties. By night and by day, in summer and winter, the general practitioner beats his rounds, till the very stones might prate of his whereabouts. Such a man is always useful, and invariably in demand. His judgment acquires ease and perspicuity from incessant experience; his manners become chastened and toned down by the sight of suffering, in which he is deeply concerned; and he has no time to play the pedant, or to affect the ostentation and folly of the age. His behaviour assumes an air of gravity, and his countenance expresses that moral grandeur which is the result of a mind engaged in the slow prosecution of truth, the steady and daily exercise of benevolence. He becomes, generally, prematurely old. His task never ceases. He seldom indulges in a holiday, either at home or abroad. Each day brings its duties, which cannot be omitted without detriment to himself or others; and, above all, the labour of every day must pay for its own expenses. As to his realizing a fortune, it is out of the question. He may save enough to keep the wolf from the door in his old age, if he live to be old; or for his family, if, as is often the case, he die early. He may catch a fever, and die; or fall into ill-health from constantly breathing the contaminated air of sick rooms and hospitals; or, in fine, he may be worn out in spite of the strongest constitution. But as to his acquiring a fortune, in the sense in which a railway contractor, or a successful stockbroker, merchant, or lawyer makes one, and counts his hundreds of thousands of pounds sterling, and maintains a London establishment and a

* Tacitus says of Petronius, the charming and well-known author of the best and the worst of satires, that, what others accomplished with trouble and hard work, he achieved with indolence and a masterly inaction.—*Annals*, xvi. 18.

country-house, and procures a commission in the army for his son, or sends him to college, and introduces his blooming daughters among the aristocracy of the land—why, fortune, in this sense of the word, is, as far as the medical man is concerned, simply ridiculous, and signifies nothing. The successful speculator never works for nothing; his prosperity is the produce of his turning everything to profit. He never deals with the poor, of whom he has no practical knowledge. He does not believe in poverty. He looks upon it as a disgrace to be poor.

Follow the medical man through yonder dismal alley, climb the creaking stairs after him, enter the dark, dirty, unfurnished room, and attend him to the bedside of that poor woman sinking from disease, and who repays him with nothing better than her prayers. Depend upon it, those prayers are written in the Book of Life, and will, on the great day of account, outshine the glitter of the luckiest millionaire upon earth. The doctor's pay is frequently nothing more substantial than a prayer; and, then, if you return home with him, you will see how modestly he is forced to live, and how hard he works in order that he may live at all.

By himself, the general practitioner cannot possibly earn more than 1500*l.* or 2000*l.* a year. With a partner, of course, it may be doubled; but by himself with a few rare exceptions, 1500*l.* a year is, perhaps, the maximum he earns. For, it must be borne in mind, that there are not more than a certain number of hours in the night and day, and a certain portion of time must be allotted to sleep and refreshment. There remain, then, not more than twelve hours for incessant occupation, and this is a much longer space of time than can be endured, or persisted in by those whose constitutions are delicate, and minds sensitive. It is not possible, therefore, to visit more than a limited number of patients every day; and considering that patients generally reside far apart from each other, and cannot be dismissed in less than a quarter or half an hour each, if the case be serious, it follows, that allowing for the distance of ground to be travelled over between house and house, not more than three patients on an average can be seen in the hour, or thirty-six patients a day. In unhealthy seasons, he may count as many as fifty on his list; but, in general, not more than thirty. Of this thirty, one-third pay

* A short period previously to the late Dr. Todd's death, I had the pleasure of meeting him at the dinner-table of a mutual friend. One of the subjects of conversation, after the ladies had retired, was the number of patients a London physician engaged in active practice could see *per hour*. Several eminent physicians present took a part in the conversation. It appeared to be the general opinion that allowing for distances, &c., *four* was the *maximum* that a physician could nothing; and the rest pay more or less according to their means; but even at its highest estimate, this will seldom produce more than 2000*l.* per annum, subject to a heavy set off on the score of business expenses. And recollect, that, to realize this sum, there must be no suspension of labour. It must be *de die in diem*—one and the same from the first of January to the thirty-first of December. Few constitutions are equal to this prolonged exertion. The health sooner or later gives way. Very few persist in carrying on so extensive a practice for many years in succession. They are forced to withdraw from it; and fortunate indeed, if, in so doing, they have something of their own to fall back upon. If not, they must go manfully forwards, and die in harness.

There are numerous instances of medical men, living on to a green old age, and cheerfully continuing the practice of their profession to the last. They have realized sufficient to enable them to keep their ground, and, when occasion requires it, to take things easily. But each healthy old man, who has weathered the storm, represents how many who have been wrecked, stranded, or gone adrift?

For the most part, young medical men commit a great mistake on first starting. They overhouse themselves. Their establishment eats them up. They have not the courage to live in a small way. Then they marry too early. It is a virtue on their part, and speaks volumes in their favour. But house expenses are certain, and professional returns uncertain. Children must be fed: servants must be paid. The wife likes the habits of a lady, decorates her rooms, and receives her friends, while her husband lives in the streets, that he may sleep in a mansion. For a mansion it is to him, that vast pile of bricks and mortar, for which he pays an enormous rental, and which he has furnished beyond his need. For the sweets of life are unknown to him. He toils only for his daily bread. He has no time for visiting. He can never receive properly. His occupations unfit him for doing so. His real post, and the one in which he shines, are the sick chamber of the wealthy and the hovels of the poor. Beyond their precincts, he is nobody. How can it be otherwise? If he is a man of fashion, he is unfit for his profession; or, if he be a philosopher, he is unfit for fashion. Add to these eccentricities and burdens, an equipage—a shining, well-turned-out equipage! This is the climax of folly that has brought many a general practitioner to the ground. It is time enough to ride in

---

visit and prescribe for in the time specified. Poor Dr. Todd came in late to the dinner, looking extremely haggard, and evidently much depressed by over work. Death appeared even then to have him in his relentless grip. The candle of life had apparently burnt to the socket.—ED.

a carriage when you have realized capital, or should be lucky enough to possess some private means of your own. Only, private means alters the question, which, as we are now looking at it, is one of pure, unaided, professional ability.

It has often occurred to us, that most medical men would be the better if they remained single. We know that it is opposed to the received opinion on the subject, and we own that it has its inconveniences. But we feel confident that, in the present state of society, in which expensive luxury forms a constant element, it is next to impossible for a general practitioner to support a proper appearance in the world from nothing more than the proceeds of his professional exertions. It is the married life that urges so many to work themselves to death. They cannot bear to see their family less than they should be. Consequently they are ever on the fret. They have no leisure to sit down and think. They cannot and must not do so; and it is owing to the cares of matrimony that many, who would otherwise have been philosophers, devoted to their profession, end by becoming nothing better than routineers or professional tradesmen. In moments of real illness and danger, the public do not ask whether the doctor rides or walks, is married or unmarried. All they require is that he should be at hand when he is wanted, and should be capable of performing all that is required of him.

There is something transcendently noble in the practice of medicine. Gold and silver are not to be compared with it, neither are they its proper reward. It is humanity in the highest meaning of the term—practical charity, which, we are assured covers a multitude of sins, "I was sick and thou visitedst me:" here is thy reward, not in this life, but the next.

A youth is dazzled by his first sight of the medical world. He sees a carriage driving by, the speed of the horses overcome with toil, and the brow of a distinguished surgeon or physician peering through the window. It is a recently created medical baronet. He is hastening to an important consultation at that great house within those portals at the corner of that street. Every one knows that its illustrious owner is at the point of death. The distinguished son of Esculapius dashes through the entry; the liveried servants receive him at the principal entrance. He crosses the marble hall, ascends the broad staircase, and enters the sick chamber. The family medical attendant is already there. The patient moans from within his canopied couch. The apartment is richly carpeted; the hangings are damask; the furniture is *en suite*, and the lofty windows are shaded by appropriate draperies. The consultation is long; the opinion grave; the fee large. The whole scene bears the impress of the grand and the imposing. It is the great world in its richest attire, convulsed in one of its saddest moments. The little moments drop away, like the sands in the hour-glass, and the great man dies. The emblazoned epitaph on his ancestral tomb in the distant village church sets forth his name and lineage, "to whom related and by whom begot."

The next year the distinguished physician dies also, from over-work ;* and is gathered not unto his fathers, but is deposited in a vault, newly excavated in the midst of strange graves, in a newly planned joint-stock cemetery. The youth does not see the issue of events, or will not see them. He only sees, in his mind's eye, the dashing carriage and the happy face within it. And certainly it is a vision never to be forgotten. Silently he breathes a fervent wish that he may live to be the same, although his favourite poet might have warned him that every scheme of happiness ends only in disappointment, as every form of life terminates only in death.†

The pageant of the world is but a dream. It moves forward in procession to a slow and stately measure, while first one and then another figure is snatched from its ranks, which are always changing, but are never changed. The phantoms keep coming and going, but the scene remains.

The English physician, both of former and recent days, has always played a prominent part in the history of medicine. He is classed among the aristocracy. Individually, he is recognised as a gentleman. His mode of education and style of thought entitle him, in a certain sense, to the homage of his brethren, and of being looked up to as a person the most proper to be consulted in cases of difficulty and danger in the last resort. All, indeed, do not succeed equally well in this respect. Many fail. The common difficulties of living beset them as well as others. A thousand trifles may be wanting to accredit the most accomplished among them to the countenance of public esteem. People are fastidious. They pick and choose as best it suits their fancy. They please themselves, they care not how, and they know not why. They are sometimes wrong, and then they resent it upon others instead of upon themselves. But, upon the whole, the *vox populi* is right in the end ; and the most deserving have seldom failed of their reward.

But the fortunate candidate for public favour is just as often the victim of success as he would have been of failure. The

---

* Worked to Death.—The carriage of the most famous physician in Dublin was waiting on Saturday last to take him on his rounds, when a strange visitor intercepted him on the way. One touch from his dart on a little cerebral vessel, and our obituary records the sudden death of Sir Henry Marsh from an apoplectic seizure.—*Lancet,* December 8th, 1860.

† The infinity of lives conducts but to death, and the infinity of wishes but to disappointment.—*Moore's Life of Byron.* Murray, 1860, p. 475.

demand upon his intellect is stupendous. It is enough to crush the strongest head, and break the stoutest heart. For he who leads must necessarily know all that is known by those whom he leads Nay, he ought to know more than they do ; for how can he advise them if he knows less? The infinite particulars of science are progressively on the increase. The particulars of yesterday are obsolete to-day, and those of to-day will be obsolete to-morrow. The leading physician must know them all. There is no use in pretending to know them. His knowledge must be absolute, and of the latest date. The feint would be detected at the first touch. It follows therefore, that he must not only be an active practitioner, but a scholar and a student besides. What an exhaustive effort! What a gigantic struggle! At home, he must study and think hard; and he no sooner steps across his threshold, than he must travel *express* all over London, and from one end of the kingdom to the other. There is no time, when he can say, Hold, enough! There is no *sick-call* that he can refuse. He can never say, No! for the first refusal from caprice or fatigue marks the summit of his reputation, and sounds the note of his decline.

Add to all this, he must sustain the character of his profession by his pen, whenever occasion calls for his doing so; and he must sustain it honourably, ably, and critically. It is expected of him that he should give to the world the fruits of his experience in the shape of a goodly volume. Most likely he lectures once or twice a week, and visits the hospital of which he is one of the staff. His day is occupied from morning till night; his hours are frequently interrupted by the calls of friendship and humanity; and society requires that he should be well versed in the political exigencies of his professional brethren, and ready to step forward and maintain their cause in the public arena of the world.

His life is a struggle against mighty odds. Ceaseless anxiety is counterbalanced by restricted pecuniary results. "Every guinea involves personal service, and every prescription is a spreading out of brains upon paper."* His life is intensified to the last degree, and consumes itself with amazing velocity. His high repute and well-earned fame weigh with a deadly pressure on his frame. Many sink under it, many more perish in the vain attempt to raise it. All aspire to it. A few sustain it.

In the character of the physician we include that of the surgeon also; for though he does not profess to be so liberally educated as the physician, yet there is no reason why he should not be so, and in fact many are. In practice he is not a whit less zealous and trustworthy than his colleague. The one solicits

* *Lancet,* December 8th, 1860.

the same public confidence, and shares the same favour, if not the same honours, as the other. In professional credit they are equals. They discharge different functions, but their object is the same.

There are few sciences in which the one and the other have not shone. There is no occasion of pestilence or national calamity in which they have failed in their duty. They are spoken of as well in the colonies as within the limits of the United Kingdom. The exceptions in which they have betrayed their trust, and tarnished the lustre of their name, are so few, that they establish the rule. Their heroism is unquestioned. They have faced the thunders of a naval engagement, or the field of battle, the trenches, and the siege; and have merited by their personal bravery the same badge of distinction as that which decorates the breasts of those by whose sides they have fought and bled.

Such men deserve much, and their pay ought to be in proportion to their talents and devotion. But is it so?

The incomes of fashionable practitioners are quoted at fabulous rates. The vulgar ear delights in marvels. It is taken for granted that they sweep the board. But so far is this from being the case, that, setting aside private fortunes with which we have no concern, the most esteemed men have come into notice late in life, and reached their professional climax in the decline of age. At the age of fifty, the late Dr. Bright was still a candidate for popular favour. And, then, as to the incomes that are made. As with the general practitioner, so with the physician, he cannot make the day longer than it is, nor pretend to more endurance than the rest of mortals. If he sees twenty patients at home in the morning, and thirty after he goes out, it is as much as he can manage, and about the same as that of his inferior medical brother whom he meets in consultation. Nor do all these fifty leave a guinea a piece behind them: some leave none: others leave more; and if the fee for a distant visit into the provinces be large, it is barely more than a compensation for business missed or lost at home, to say nothing of the bodily fatigue undergone in a long and hasty journey, and the responsibility incurred by delivering judgment on a case from which there ought to be no appeal. *Five thousand pounds* a year, or *three thousand pounds* at least, is not more than is requisite to maintain a first-rate London establishment in necessary working order for an extensive consulting practice. Doubtless, much larger incomes than this are made; but we ask, how many surgeons and physicians of note are making as much as this, or as little?

The most painful portion in the history of a physician's life are the early years of his commencement, which are passed out of sight, and in straitened circumstances. The bitterness of this period darkens the spirits, and throws a deep shadow across the brightest futurity.

Can civilization proceed at its present speed and intensity? Can life subsist under it? Will it not eventually wear itself out? We think not. Facts declare themselves in our favour. Longevity is upon the increase: statistics prove it. The health of the community is good. Where seven or eight died formerly, only two die now; where pestilence once raged, it is now extinct.* The race improves. Stature and strength are greater than they used to be. Free institutions, freedom of thought, and religious as well as civil liberty, enlarge the sphere of vision, extend the faculties of the mind, and multiply the resources of happiness and enjoyment. Cloudy as the political horizon may seem, yet the gleam of light along its margin is bright and encouraging. The privileges of mankind are more fully appreciated, and less likely to be invaded or oppressed by domestic or foreign aggression than at any former period of the world's history. Steam and the electric wire are working wonders in a way that cannot be disputed. Their beneficial operations will not cease. They are public property; and their utility is too well understood ever to allow of their being laid aside and forgotten.

In regard to ourselves, as medical men, is the prospect less cheering than we could wish? At present we are overtasked, or at least they are upon whom the world has bestowed the meed of merit. But was it ever otherwise? Must it not continue to be so? The pet of the public lies at the mercy of an exacting patron. A favourite is never his own master; and when we complain of having more favour than enough, let us not forget those who repine because they are neglected, and get less than they want. In a certain sense the evil is irremediable. For

> "Some must laugh, while some must weep;
> So trips the world away."

And the ambitious will suffer, as well as achieve, the most. Except in a few favoured instances, "the cool, sequestered vale of life" is out of the question; and none but the old, the disabled, or the timid, would covet the seclusion.

---

* In the seventeenth century, the mortality was at the rate of seven per cent. on an average during twenty years. If the mortality of London had been at the same ratio in the last year (1859), instead of 61,617, about 194,204 deaths would have been registered. In 1665 nearly a third of the population perished by the plague.—*Registrar-General's Summary of the year* 1859, p. vii.

